# Editorial: Reviews in: stroke

**DOI:** 10.3389/fneur.2024.1364970

**Published:** 2024-02-13

**Authors:** Bernhard Sehm, Kristian Barlinn, Eduardo Candelario-Jalil, Krishna Kumar Veeravalli, Chunping Ni, Jun Yao

**Affiliations:** ^1^Department of Neurology, University Hospital Halle, Halle (Saale), Germany; ^2^Department of Neurology, Max Planck Institute for Human Cognitive and Brain Sciences, Leipzig, Germany; ^3^University Hospital Carl Gustav Carus, Dresden, Germany; ^4^University of Florida, Gainesville, FL, United States; ^5^Departments of Cancer Biology and Pharmacology, Neurosurgery, Pediatrics, and Neurology, University of Illinois College of Medicine Peoria, Peoria, IL, United States; ^6^School of Nursing, The Fourth Military Medical University, Xi'an, Shaanxi, China; ^7^Physical Therapy and Human Movement Sciences Department, Northwestern University, Evanston, IL, United States

**Keywords:** stroke, acute, diagnostic, etiology, neuroimaging, intervention, rehabilitation

Stroke constitutes a significant global health challenge, with 12.2 million new strokes per year worldwide, a mortality of 6.5 million, and a cumulative disability of 143 million disability-adjusted life years [DALYs; (1)]. To confront this challenge, the clinical and scientific community is dedicated to addressing various aspects, including primary and secondary prevention, acute stroke care, biomarkers, patient stratification, and neuroplasticity and rehabilitation. The approaches involved encompass both clinical randomized trials and hypothesis-driven or exploratory research.

The global efforts in stroke research result in a substantial number of publications per year, as evidenced by a swift exploration of the PubMed database using the search term “stroke,” which yielded over 34,000 references in the year 2022 only. This deluge of literature highlights the necessity for meta-analyses and systematic reviews, which aggregate and synthesize a large body of evidence from multiple studies using comprehensive and systematic approaches. We, therefore, have collaborated to curate this Research Topic, with the following objectives: (i) increasing the statistical power by combining data from multiple clinical and preclinical studies and thereby helping to estimate the robustness of an effect; (ii) enhancing the generalizability of findings and fostering the application of preclinical research insights into broader clinical applications; and (iii) exploring subgroups or moderating factors for an observed effect and thus help to personalize treatment strategies.

The Research Topic “*Reviews in: stroke*” includes 25 articles that cover a variety of topics around stroke. One of the major emphases is on acute stroke diagnostics and treatment. We capture current topics like the impact of COVID-19 on acute ischemic stroke care (Stuckart et al.) or the use of telemedicine in acute stroke and its diagnostic accuracy (Poongkunran et al.). Several articles provide insight into neuroimaging (Zheng et al.; Huang et al.; Yoshimoto; Cheng et al.) and serum markers (Liu Y. et al.) that might help with patient selection and treatment monitoring in acute stroke. Other articles look at new treatment options for both ischemic and hemorrhagic stroke [(2); Zheng et al.; Fu et al.].

A further focus of this Research Topic is on risk factors and stroke etiology and addresses questions such as: what is the impact of inflammatory processes on the risk for atherosclerosis and stroke (Wang L. et al.; Jia et al.; Xie et al.; Pinzon et al.)? Can we identify risk factors for in-stent stenosis after intracranial stenting (Wang N. et al.)? And what do we know about perioperative stroke, an entity that is clinically relevant but only marginally addressed in the literature (Ji et al.), and the etiology of cervical artery dissection (Gunduz et al.)?

The third emphasis is on stroke rehabilitation and covers therapeutic interventions such as constrained-induced movement therapy (Cui et al.), balance training (Zhang et al.), and interventions in post-stroke dysphagia (Liu J. et al.). A further article covers the use of AI for the prediction of post-stroke cognitive deficits (Li et al.).

Taken together, this Research Topic provides a variety of topics related to clinical and preclinical stroke research with the common aim of synthesizing the evidence using systematic reviews and meta-analyses. We hope that it will help clinicians and scientists in the field of stroke and foster translational progress.

## Author contributions

BS: Writing – original draft. KB: Writing – review & editing. EC-J: Writing – review & editing. KKV: Writing – review & editing. JY: Writing – review & editing. CN: Supervision, Writing – original draft, Writing – review & editing.

## References

[1] Feigin VL Brainin M Norrving B Martins S Sacco RL Hacke WL. World Stroke Organization (WSO): global stroke fact sheet 2022. Int J Stroke. (2022) 17:18–29. 10.1177/17474930211065917 34986727

[2] Haiyan. Can the combination of antiplatelet or alteplase thrombolytic therapy with argatroban benefit patients suffering from acute stroke? A systematic review, meta-analysis, and meta-regression. 10.1371/journal.pone.0298226 PMC10898750 38412157

